# Rare Variants May Influence Disease Risk and Clinical Features in Sporadic Late-Onset Chinese Parkinson’s Disease Patients

**DOI:** 10.3390/ijms27177653

**Published:** 2026-08-26

**Authors:** Ryan Wui-Hang Ho, Zewei Xiong, Rachel Cheuk-Nam Lo, Huifang Liu, Philip Wing-Lok Ho, Pak-Chung Sham, Shu-Leong Ho, Shirley Yin-Yu Pang

**Affiliations:** 1 Division of Neurology, Department of Medicine, Queen Mary Hospital, The University of Hong Kong, Hong Kong SAR, China; ryanhowh@connect.hku.hk (R.W.-H.H.); slho@hku.hk (S.-L.H.); 2 Department of Psychiatry, Li Ka Shing Faculty of Medicine, The University of Hong Kong, Hong Kong SAR, Chinapcsham@hku.hk (P.-C.S.); 3 Department of Rehabilitation Sciences, Faculty of Health and Social Sciences, The Hong Kong Polytechnic University, Hong Kong SAR, China; 4 Mental Health Research Centre, PolyU Academy for Interdisciplinary Research, The Hong Kong Polytechnic University, Hong Kong SAR, China; 5 Research Institute for Smart Ageing, The Hong Kong Polytechnic University, Hong Kong SAR, China; 6 Centre for PanorOmic Sciences, Li Ka Shing Faculty of Medicine, The University of Hong Kong, Hong Kong SAR, China; 7 State Key Laboratory of Brain and Cognitive Sciences, The University of Hong Kong, Hong Kong SAR, China

**Keywords:** Parkinson’s, genetics, genotype–phenotype, rare variants

## Abstract

Heritability in Parkinson’s disease (PD) may be driven by rare variants (RVs), but genetic data for Chinese cohorts remain limited. We investigated the relationship between RVs and the risk and phenotype of PD by performing targeted exome sequencing of 29 PD candidate genes in 311 late-onset, sporadic Chinese PD patients and 699 local controls. A total of 174 RVs (that were likely deleterious using in silico prediction tools) were identified in 27 of the 29 genes sequenced. Mean RV burden was higher (1.178 vs. 0.478, *p* = 1.26 × 10^−24^) and *HLA-DRB5* p.Val104ArgfsTer26 was enriched in PD patients (0.207 vs. 0.000, *p* < 0.0003) compared with controls. Gene-based RV carrier status in PD patients was then examined for correlation with phenotypic characteristics. The RV carrier status of *LRRK2* and *HLA-DRB5* was associated with tremor, *SREBF1* with gait disturbance, and *SYNJ1* and *SCARB2* with motor fluctuations. Earlier onset age and neuropsychiatric symptoms were associated with *GBA1* variants. Olfactory deficit, autonomic disturbance, anxiety and depression were associated with variants in *SLC44A1*, *HLA-DRB5*, and *SCARB2* respectively. Our results demonstrated a prominent RV burden in Chinese PD and illustrated the importance of genetic influence on the risk and phenotype of sporadic PD. Further studies are warranted to correlate these genes with putative pathogenic pathways.

## 1. Introduction

Parkinson’s disease (PD) is characterized pathologically by loss of dopaminergic neurons in the substantia nigra and accumulation of intracellular Lewy bodies. Clinically, PD is characterized by motor features of bradykinesia, rigidity and rest tremor, and non-motor features including hyposmia, sleep disturbances and cognitive impairment. PD is a heterogeneous disorder: motor and non-motor symptoms manifest and progress in varying combinations and rates in different patients.

In line with the clinical heterogeneity, the etiology of PD involves a complex interplay of aging, genetics and environmental factors [1]. Since the discovery of the first causative gene, *SNCA*, studies on monogenic PD families have revealed pathogenic rare variants in many genes (e.g., *PRKN*, *DJ1*, *PINK1*) [2]; however, these mutations remain rare in the wider PD population. The subsequent advent of genome-wide association studies (GWASs) allowed for the identification of common risk variants for apparently sporadic PD, and many loci were located in monogenic PD genes (e.g., *LRRK2* [2]). Despite these advances, significant gaps remain. For example, heritability of PD was estimated to be 22–27% [3] but GWAS risk loci accounted for only 16–36% of this heritability [3]. The missing heritability is likely due to rare variants, epigenetics, and structural variants [4], many of which remain unknown and uncataloged. Furthermore, although subsequent GWASs have yielded datasets with increasingly large sample sizes, these cohorts often lack substantial clinical data to study disease characteristics and progression. They also lack genetic diversity as the participants are predominantly of European ancestry. Indeed, some of the missing heritability of PD may be discovered through the study of non-European populations [5].

In recent years, various candidate genes have been mapped to putative biological pathways in PD. Mutant *SNCA* produces α-synuclein that is more liable to pathological misfolding and aggregation, giving rise to Lewy bodies and their associated neurotoxicity. Variants in genes such as *GBA1*, *LRRK2*, *SCARB2*, *RAB29* have been linked to disruption in the autophagy–lysosome pathway, leading to impaired clearance of α-synuclein and other faulty proteins [6]. Other PD-associated genes have been mapped to PD pathogenic pathways including mitochondrial function (e.g., *PINK1*, *PRKN*, *DJ-1*, *ATP13A2*) [7] and immune function (e.g., *HLA-DRB1*) [8]. Genes have also been identified in PD risk GWASs (e.g., *MAPT*, *CTSB*, *SV2C*) [9,10], age of onset GWASs (e.g., *TMEM175*) [11] and progression GWASs (e.g., *SLC44A1*, *ADRA2A*) [12]. In addition to these genes, in this study we added 14 well-validated PD causal genes [11] to generate a final panel of 29 genes (Table 1). We aim to identify rare variants among these genes in a cohort of largely sporadic and late-onset Chinese PD patients and further investigate their correlation with the risk and clinical characteristics of PD.

## 2. Results

### 2.1. Demographic and Clinical Features of Patients

A total of 311 PD patients were recruited from the neurology clinic at Queen Mary Hospital and the movement disorder clinic at Tung Wah Hospital, in Hong Kong, with a mean (SD) age of 66.54 (±8.75) years (Table 2). The mean age of onset was 60.51 (±9.75) years and mean disease duration was 6.48 (±5.12) years. There was a slight male predominance (54.6%). A small proportion (4.1%) reported positive family history, with a parent or sibling affected with PD. Similarly, only 16 (5.14%) patients had early (<45 years old) onset. Most patients had mild/moderate PD: 54.6% at Hoehn and Yahr stage 2 and 23.1% at stage 3. The mean MoCA score was 23.25 (±4.99), and their motor and non-motor assessment scores are shown in Table 3. Mean levodopa equivalent daily dose was 456mg (Table 3).

### 2.2. Rare Variant Identification

A total of 174 rare variants were identified in 27 of the 29 genes sequenced (Supplementary Tables S1 and S2), 48 of which have not previously been reported in gnomAD East Asian population. No rare variants were found in *VPS35* and *UCHL1* in patients or controls. The most frequently found variant in PD patients was *HLA-DRB1 p.Gln99ArgfsTer31* with an allele frequency of 0.249, followed by *HLA-DRB5 p.Thr214MetfsTer15* at 0.225. *VPS13C* had the highest number of rare variants identified (14 of 33 were found only in PD patients). On the other hand, of the 12 rare variants found in *PLA2G6*, 10 were observed only in controls. Twenty-three rare variants occurring in nine genes have been associated with PD or related disorders on ClinVar [13] (Table 4).

### 2.3. Effect of Rare Variants on Risk of PD

To study the effect of rare variants on the risk of PD, allele frequencies of identified rare variants were compared between PD and controls. Among the 156 rare variants with adequate sequencing coverage in both the PD and control groups, one was found to be significantly associated with PD after correcting for multiple comparisons: *HLA-DRB5* p.Val104ArgfsTer26 with a frequency of 0.207 in patients (*p* < 0.0003). This variant was rare in the gnomAD East Asian population (MAF = 0.0004501) and was not found in local controls.

The mean rare variant burden in PD patients was significantly higher than that of controls (1.18 vs. 0.478, *p* = 1.26 × 10^−24^). Furthermore, a greater proportion of control subjects carried zero rare variants (63% vs. 29%), whereas significantly more PD patients carried two or more variants (Table 5).

### 2.4. Effect of Rare Variant Burden on Clinical Features

We then examined the effect of rare variants on clinical features of PD. A total of 141 rare variants in 25 genes with adequate sequencing quality in the PD group were included in the analysis for phenotype correlation. Rare variants in each gene were examined using SKAT (permutated *p*-values corrected for multiple comparisons) for association with clinical features including age of onset, motor features (by Movement Disorder Society-sponsored Revision of Unified Parkinson’s Disease Rating Scale (MDS-UPDRS)), non-motor symptom burden (by Non-Motor Symptoms Questionnaire (NMSQuest)) and cognitive function (by Hong Kong Montreal Cognitive Assessment (HK-MoCA)), adjusting for sex, age, disease duration and levodopa equivalent daily dose. No significant association was observed between the rare variant burden in any gene and the non-motor symptoms of apathy, cognitive deficits and psychosis, dopamine dysregulation, MoCA score, and motor symptoms of bradykinesia and rigidity. Significant results are shown in Table 6.

## 3. Discussion

In this study, we systematically analyzed rare variants in 29 PD-related genes and their associations with the risk and phenotype of PD in a cohort of Chinese PD patients. Although large meta-analyses have been carried out to elucidate the effect of rare variants on PD risk [14], they were mostly performed on subjects of European ancestry, and ours is among the few studies [15,16,17] evaluating an Asian population.

We found a higher aggregate rare variant burden in PD patients compared to controls (1.18 vs. 0.478, *p* = 1.26 × 10^−24^), and more PD patients than controls had two or more rare variants (34% vs. 9%). An Indian study [16] also found higher aggregate protein-altering rare variant burden in PD patients than controls (0.91 vs. 0.51) in 20 PD-related genes. A Chinese study [17] found that more PD patients carried potentially deleterious rare variants than controls (14.1% vs. 3.5%) in 12 genes. Other studies, however, failed to find an overall rare variant enrichment in PD-related genes among patients [15,18]. These inconsistencies must be interpreted in light of methodological heterogeneity. Firstly, the criteria utilized for gene selection varied across studies. Some focused on Mendelian genes from familial PD [17] while others focused only on genes in proximity to GWAS susceptibility loci [19,20]. Others, similar to our study, covered a mixture of both [15,18]. Variant filtering also differed. Some studies analyzed the overall burden of all non-synonymous coding variants [15,18,19], potentially diluting the effect by including bystander low-impact variants. Our finding of a higher rare variant burden in PD patients was consistent with other studies filtering for high-impact rare variants only using in silico prediction tools [17,20]. Moreover, some previous studies [16,17] were done on cohorts with large proportions of early-onset PD (EOPD) or with family history, potentially enriching highly penetrant variants. Our study showed a significantly higher aggregate rare variant burden even within our cohort of largely sporadic, late-onset Chinese PD patients (5% young onset, 4% with family history, mean age of onset 60.5). We also identified rare variants across a spectrum ranging from well-validated Mendelian ‘familial PD’ genes to GWAS-linked genes with few prior reports in familial PD (e.g., *HLA-DRB5*). Our observations, together with previous findings from many GWASs which detected common risk variants at known Mendelian gene loci [4], challenged the conventional dichotomization into highly heritable, ‘familial PD’ arising from rare deleterious variants at a handful of Mendelian genes and less heritable, ‘sporadic PD’ accounted for by low-effect-size common variants in a large number of ‘risk’ genes (common-disease common-variant hypothesis). These findings support the conceptualization of PD as a multifactorial disease in which genetic predisposition plays a major role in influencing individual susceptibility to other potentially pathogenic factors such as aging and environmental factors [1].

Across the panel of 29 PD-related genes we analyzed, rare variants (174 in total) were found in all genes except *VPS35* and *UCHL1*, suggesting that the genes on our gene list were highly relevant for Chinese PD patients. The gene with the highest number of rare variants found (33 rare variants, of which 14 were only found in PD patients) was *VPS13C*, a PD risk GWAS hit [21] that was also identified in EOPD [22]. A significant proportion (48 (27.6%)) of the variants found in this gene were novel in the East Asian population and only a minority (23 (13.2%)) of them were previously described to be associated with parkinsonism and related disorders, again highlighting that a significant proportion of PD genetic susceptibility has yet to be explored or cataloged. Furthermore, a large proportion of PD patients were found to carry multiple rare variants. For instance, 34% of PD patients carried two or more rare variants, while five (1.6%) carried more than three rare variants. Since the most common rare variant we identified in our patients was *HLA-DRB5* p.Val104ArgfsTer26 (MAF = 0.207), it is not surprising that the most common combination of rare variants is homozygosity of *HLA-DRB5* p.Val104ArgfsTer26 (eight patients), followed by that with *MAPT* p.Gln230Arg (seven patients) and that with *VPS13C* p. Arg800Ser (five patients). Whether these co-occurrences of rare variants interact and further influence the clinical phenotype warrants further investigations.

*HLA-DRB5* p.Val104ArgfsTer26, the most commonly found rare variant in our PD cohort, was also the only rare variant found to be significantly enriched in PD patients at the variant level. *HLA-DRB1* and *-DRB5* encode the β-chain of the heterodimeric HLA-DR major histocompatibility complex (MHC) class II receptor in antigen-presenting cells (APCs) and have been linked to a variety of autoimmune diseases [8]. Mounting evidence supported a role for the immune system in PD pathogenesis [8]. GWASs identified single nucleotide polymorphisms (SNPs) in *HLA-DRA* [23] and *-DRB5* loci [3] correlating with the risk of sporadic PD, with subsequent HLA allele imputation studies pinpointing HLA-DRB1*04 as a protective allele against PD [24,25]. MHC-II-dependent alpha-synuclein-induced activation of microglia, the resident APC of the brain, was associated with dopaminergic neuron degeneration in animal models [26]. Variable affinity by MHC-II molecules to alpha-synuclein epitopes encoded by its genetic variability [25] may plausibly lead to a variable immune response to alpha-synuclein, thus influencing PD risks. Furthermore, *HLA-DRB5* has been shown as a risk locus for ulcerative colitis and it has been postulated that the pathological process of PD may originate in the gastrointestinal tract (the “gut–brain axis theory”) where a chronically inflamed gut may trigger alpha-synuclein deposition and increase permeability of the blood–brain barrier leading to neuroinflammation [27]. We identified ten and seven rare variants (minor allele frequency (MAF) < 0.05 in gnomAD) in *HLA-DRB1* and -*DRB5* respectively, and ten of the 17 were relatively common (frequency > 0.1) in PD subjects. Unfortunately, insufficient sequencing coverage in the control group precluded direct comparison for a majority of these rare variants, but we did identify one *HLA-DR5* frameshift variant, p.Val104ArgfsTer26, that is significantly enriched in PD patients compared to controls. It should be noted that *HLA* loci are often challenging to study because of the especially complex linkage disequilibrium among different HLA loci [24], considerable interracial variability [25], and the additional confounding effect by regulatory non-coding variants influencing expression of downstream HLA alleles [28]. Nevertheless, our preliminary findings support the role of *HLA-DR* β-chain in PD genetic susceptibility, and provide circumstantial supportive evidence of the involvement of the immune system in PD pathogenesis.

Several genes in our gene list are of special interest. Among the most commonly mutated genes in PD [1], *leucine-rich repeat kinase 2* (*LRRK2*) was consistently reported in both monogenic families and GWASs [3,9], supporting its pleomorphic role in monogenic and sporadic PD. *LRRK2* was linked to intracellular trafficking, as well as endolysosomal and mitochondrial functions [29], and may play a role in PD pathogenesis via a toxic gain-of-function mechanism (increased kinase activity) [1]. The most common and perhaps the best-characterized *LRRK2* variant, G2019S, was not found in our cohort, corroborating prior reports of its rarity in Chinese populations [30]. On the other hand, R1441C, a variant also mostly reported in European patients [31] and rarely in Chinese PD patients [32,33], was found in one PD patient and no controls. R1441C was shown to disrupt *LRRK2*’s GTPase activity in vitro [34], interact with synaptic vesicle proteins and cause motor deficits in animals [35]. Our patient with the R1441C mutation did not have any family history of PD and had mild motor and non-motor symptoms, consistent with other reports of LRRK2-PD having a more benign disease course [36]. Three patients (and five controls) were noted to carry A419V. Although meta-analyses suggested an epidemiological link with PD [31], its pathogenicity has been disputed [37]. Our results confirmed previous observations that known pathogenic *LRRK2* mutations are rare in Chinese patients.

*Glucocerebrosidase 1* (*GBA1*) mutations were first described in Gaucher disease (GD), where mutant glucocerebrosidase with reduced enzyme activity causes a multisystem disease with variable neurological involvement. The postulation of a link between *GBA1* and PD first arose when some non-neuronopathic GD patients were noted to have parkinsonism. Subsequent studies have revealed that *GBA1* mutations occurred in 7–15% of PD patients [38], making it the most important risk gene in PD [1]. Nine *GBA1* variants were identified in ten patients in our cohort and all except one (R202Q, found in two controls) were present only in PD patients. R202Q has also been described in Chinese PD patients [38,39], but its pathogenicity is difficult to establish as it is also relatively common (0.296%) in East Asians. Although we did not identify any patients with common *GBA* pathogenic variants such as N370S, E326K, or L444P [40] (L444P is reported to be common in Chinese populations [1]), three known pathogenic variants, D448H, R87W and c.762-1G>C, were identified in our cohort. Among the four remaining variants, D63N (one patient) was previously reported only in type 1 GD patients [41] and the other three were novel. In line with the literature [38], *GBA1* was associated with an earlier onset age in our cohort.

*PLA2G6* encodes for calcium-independent phospholipase A_2_β (iPLA2_2_β), a widely expressed phospholipase that was thought to play an important role in synaptic transmission and mitochondrial function [42]. Mutant *PLA2G6* is associated with a wide range of clinical phenotypes (collectively known as PLA2G6-associated neurodegeneration (PLAN)) [42], and its identification in early-onset dystonia–parkinsonism led to its designation as the *PARK14* locus [43,44]. Subsequent studies also identified *PLA2G6* mutations in late-onset PD [45], though its association with sporadic PD was brought into question by a meta-analysis failing to find a significant association between previously reported *PLA2G6* polymorphisms and PD risk [46]. Ten of the 12 *PLA2G6* variants in our cohort were only found in controls, including D331Y [43,47] and c.1427+1G>A [47] previously reported in early-onset parkinsonism. P806R was found in seven PD patients and six controls. No significant enrichment was seen after correcting for multiple comparisons, similar to a Japanese study [45]. One PD patient carried R53H along with three controls. In summary, our finding of the *PLA2G6* rare variant being less common in Chinese PD patients than in controls did not support *PLA2G6* as a risk gene for sporadic PD in Chinese populations and might even suggest a potential protective role.

We also explored the genotype–phenotype association in our cohort based on rare variant carriage of each tested gene. So far, few studies have focused on the role of rare variants (apart from well-known genes such as *GBA1*) in influencing the clinical phenotype of sporadic PD. Risk loci identified in GWASs on the age of onset [11] and rate of progression [12] only partially overlapped with those in classical PD risk GWASs [4], suggesting that genetic factors in determining the risk of PD may be different from those influencing phenotypic features, highlighting the complex, multifaceted nature of genotype–phenotype correlation in PD and, by extension, their underlying biological pathways. As all our patients were assessed with standardized protocols, we were able to obtain detailed phenotypic data such as motor scores and non-motor symptom burden, making it possible to study the effects of rare variants on clinical features. Specifically, we assessed motor symptoms and their severity (rigidity, bradykinesia, gait, and tremor) using Part III of MDS-UPDRS, and motor complications (motor fluctuation and dyskinesia) using Part IV of MDS-UPDRS. Burden of non-motor symptoms was assessed using NMSQuest which included 30 items encompassing different domains such as neuropsychiatric symptoms, sleep disorders, autonomic symptoms and sensory symptoms. Limited by our relatively small (albeit deeply phenotyped) sample size of 311 patients, our exploratory efforts nevertheless yielded the interesting observation that some PD-related genes were associated with different aspects of the PD phenotype. In particular, motor features of tremor and gait, motor complications of fluctuation and dyskinesia, and non-motor symptoms in the neuropsychiatric domain, anxiety and depression, olfactory deficit, sleep disorders and autonomic dysfunction showed significant correlation with some of the PD-related genes we studied (Table 6).

Motor manifestations in PD can be heterogenous. A European GWAS found that a polygenic risk score predicts PD motor subtype, though no associations with individual genes were found [48]. In our cohort, *LRRK2* rare variant carriers scored higher on the tremor sub-score of UPDRS Part III compared to other PD patients. This suggestion of a tremor-predominant phenotype in *LRRK2*-PD is consistent with some [36] but not all [49] prior studies. In contrast, *HLA-DRB5* carriers had lower tremor scores. Interestingly, there were reports of a positive correlation between motor symptom severity and peripheral cytokine production [50], though none specifically studied their association with tremor. This suggested that the immune system may play a role in determining the phenotype. *SREBF1*, another PD risk GWAS hit [51] encoding for a transcription factor in the lipogenesis pathway linked to mitophagy [52], was associated with worse gait disturbance. Motor fluctuations were more common in *SYNJ1* and *SCARB2* carriers, a PD risk [51] and age-of-onset [11] GWAS hit. Notably, patients with early-onset monogenic *SYNJ1* [53] were also reported to exhibit prominent levodopa-related dyskinesias, suggesting that it may be involved in biologic pathways of dyskinesia.

Non-motor symptoms (NMSs) are ubiquitous in PD and may be an even more important contributor to disability than motor symptoms. The onset of autonomic dysfunction, hyposmia and sleep disorders often predate motor parkinsonism by years, representing a prodromal phase of PD. Understanding their genetic basis may provide important insights to the upstream pre-symptomatic stages of the PD pathogenesis process. In our cohort, *SLC44A1* carriers, a GWAS hit linked to the PD progression rate [12], reported more severe olfactory deficits. Autonomic dysfunction was found to be more prominent in *HLA-DRB5* carriers. Additionally, neuropsychiatric disturbances including mood disorders, psychosis and cognitive impairment also feature prominently among PD patients and are highly debilitating. Consistent with the past literature [38], *GBA* carriers in our study scored higher on a combined neuropsychiatric disturbance score (including NMSQuest items on memory impairment, psychosis, apathy, anxiety and depression). Anxiety and depression scores were higher in carriers of *SCARB2*. Another often neglected and yet important NMS is pain. In our cohort, higher pain scores were seen in carriers of *LRRK2*, *SYNJ1* and *GPNMB* (a GWAS risk locus [3] shown to interact with alpha-synuclein in vitro [54]). Little is known from the literature on the genetic basis of pain in sporadic PD, but there were reports that PD patients with autosomal dominant *LRRK2* mutations may experience painful ‘off’-state dystonias [55]. With the increasing recognition of NMSs as an integral part of PD symptomatology, our findings, though largely exploratory in nature, provide important clues to a possible genetic basis in a specific NMS phenotype in PD. Further investigations into the underlying biological pathways may identify specific treatments to address this unmet need of debilitating non-motor symptoms in PD.

This study has several limitations. Firstly, the modest sample size restricted the number of rare variant carriers identified. Consequently, the study may have been underpowered to detect smaller effect sizes of certain RVs, whilst simultaneously leaving the analysis susceptible to false positives from random effects and over-interpretation of apparent associations. In particular, the exploratory analysis on the association between gene-based carrier status and phenotypic features were based on a small number of patients only. Our cohort was predominantly Chinese, limiting generalizability to other ethnic populations. However, our study also supplemented current knowledge of the PD genetic landscape which was largely derived from Caucasians. Our control group was based on a volunteer-based community cohort, which might plausibly contain subjects with undiagnosed PD or prodromal PD. Nevertheless, the effect of this should be negligible with local PD prevalence at 0.186% (~1/538) only [56]. Mapping the quality of some variants of interest (e.g., *LRRK2* G2385R) in our cohort did not pass the QC process, and hence these were not reported, meaning that some potential associations with the PD risk and phenotype may be missed. In particular, the complexity of *HLA* gene structures resulted in coverage limitations that may undermine the reliability of our findings in *HLA-DRB5*. We also did not include detailed assessment of the severity and frequency of each NMS specifically.

## 4. Materials and Methods

### 4.1. Subjects

Patients aged 19–80 years who were diagnosed with PD according to the United Kingdom PD Society Brain Bank clinical diagnostic criteria [57] were recruited from the neurology clinic at Queen Mary Hospital and the movement disorder clinic at Tung Wah Hospital in Hong Kong between March 2020 and September 2022. Clinical information including age, sex, age at onset, disease duration, family history of PD, levodopa equivalent daily dose, and Hoehn & Yahr staging were recorded.

### 4.2. Controls

A total of 699 exomes from volunteer-based healthy controls that we have previously described were used for case–control association tests [58]. These volunteers were considered healthy controls based on their own reporting and were not evaluated specifically for symptoms and signs of PD.

### 4.3. Clinical Assessment

Motor features were assessed using the Movement Disorder Society-sponsored Revision of Unified Parkinson’s Disease Rating Scale (MDS-UPDRS) [59]. Sub-scores from different items of MDS-UPDRS Parts III and IV were calculated to assess specific motor features: rigidity (items 3.3a–e), bradykinesia (items 3.4–3.8, 3.14), gait (items 3.9–3.13), tremor (items 3.15–3.18), dyskinesia (items 4.1–4.2), motor fluctuations (items 4.3–4.5).

Non-motor features were assessed using the Non-Motor Symptoms Questionnaire (NMSQuest) [60]. Scores for various domains of non-motor symptoms were calculated: neuropsychiatric (items 12–14, 16–17, 30), anxiety/depression (items 16, 17), apathy (item 13), cognitive deficits and psychosis (items 12, 14, 30), olfactory deficits (item 2), pain (item 10), sleep disorders (items 22–26), and autonomic dysfunction (items 1, 3–9, 19–20, 27–28). Cognitive function was assessed using the Hong Kong Montreal Cognitive Assessment (HK-MoCA) [61].

Permission was obtained from the International Parkinson and Movement Disorder Society for the use of MDS-UPDRS and NMSQuest.

### 4.4. Patient Consent

This study was approved by the Hong Kong Hospital Authority/Hong Kong West Cluster Institutional Review Board Ethics Committee and all participants gave written informed consent.

### 4.5. PD Gene List

Twenty-nine protein-coding genes were sequenced (Table 1). These include known causal genes for PD [2], possible candidate PD genes [6,24,54] and genes identified in GWASs [9,10,11,12,62].

### 4.6. Sequencing, Genotyping and Variant Calling

Whole-exome sequencing was previously performed on 699 exomes from volunteer-based healthy controls in Hong Kong using Illumina TruSep Exome capture kits with 48× coverage overall.

Targeted-exon sequencing was performed on 311 PD patients using Nimblegen SeqCap EZ KAPA HyperPrep Kits. The reads were mapped to the Human Genome hg38 reference genome with Burrows–Wheeler transform software (v0.7.17) [63]. The Picard toolkit (v2.18.9) [64] was adopted for lifting reads to Human Genome hg19 as well as marking and removing duplicated reads. The Genome Analysis Toolkit (GATK; v3.8.1.0) [65] best practices pipeline was adopted for local realignment, base quality recalibration and variant calling. Variants in these analyses passed the following quality control (QC) steps: genotype missing rate < 0.2 and QD > 2.0. Additional QC steps applied for SNPs: FS < 60.0; MQ > 20.0; MQRankSum > −12.5 and ReadPosRankSum > −8.0, and for INDELs: FS < 200.0 and ReadPosRankSum > −20.0.

Variants were annotated using the Ensembl Variant Effect Predictor package (VEP; v93.2) [66]. The Combined Annotation Dependent Depletion (CADD, which assesses the deleteriousness of single-nucleotide and indel variants) [67] and Loss-of-Function Transcript Effect Estimator (LOFTEE, which assesses stop-gain, frameshift and splice-site disrupting variants) [68] VEP plugins were used. Variants in rare variant association analyses were filtered by the following two steps: (1) minor allele frequency (MAF) < 0.05 or not reported (‘novel’) in East Asian population from The Genome Aggregation Database (gnomAD) v.2.0.1; and (2) ‘high’ variant impact defined by LOFTEE or missense variant with CADD > 15.

### 4.7. Statistical Analysis

Rare variant burden in cases and controls was compared using the chi-squared test and Student’s *t*-test. Rare variant association tests at gene and gene-set levels were performed by RVTEST (v2.1.0) [69] between case/control and case/clinical scales. The Sequencing Kernel Association Test (SKAT) was used to evaluate rare variant burden association. Tests between case/control were adjusted for sex while tests between case/clinical scales were adjusted for sex, age, disease duration and levodopa equivalent daily dose.

## 5. Conclusions

This study showed that in a cohort of sporadic late-onset Chinese PD patients, the aggregate rare variant burden in 29 PD-related genes was significantly higher compared to healthy controls, affirming the notion that most PD cases are genetically influenced. After correcting for multiple comparisons, *HLA-DRB5* p.Val104ArgfsTer26 is the only rare variant that showed significant enrichment in PD patients, highlighting the role of the adaptive immune system on PD pathogenesis. Gene-based analysis revealed that rare variant burden in a number of PD-related genes may influence the motor and non-motor features of PD. Corroborating with previous observations from GWASs, genes influencing PD risk may be different from genes influencing specific motor and non-motor features, suggesting different pathogenic pathways driving disease onset and progression. Our results illustrated the important but probably under-recognized contribution by rare variants towards sporadic, late-onset PD, and supplemented the existing literature which is largely derived from Caucasian populations with information on the genetics of PD in Chinese populations. Importantly, the current treatment strategy for PD remains symptomatic based largely on dopamine replacement, which is known to be ineffective for many non-motor symptoms. Findings of our study on the impact of some genes on gait disturbance, pain and neuropsychiatric symptoms suggest the possibility for specific treatment of these troublesome symptoms by targeting these genes and their biological pathways.

## Figures and Tables

**Table 1:** List of candidate genes included in this study.

Causal PD Genes	Genes Identified from GWAS	PD Candidate Genes
*Synuclein alpha (SNCA)* *Parkin RBR E3 ubiquitin protein ligase (PRKN)* *Oncogene DJ1 (DJ1)* *PTEN-induced kinase 1 (PINK1)* *DNA Polymerase Gamm, catalytic subunit (POLG)* *ATPase 13A2 (ATP13A2)* *F-box only protein 7 (FBXO7)* *Glucosylceramidase beta 1 (GBA1)* *Phospholipase A2, group VI (PLA2G6)* *VPS35 retromer complex component (VPS35)* *DNAJ/HSP40 homolog, subfamily C, member 6 (DNAJC6)* *Synaptojanin 1 (SYNJ1)* *Vacuolar protein sorting 13 homolog C (VPS13C)* *Leucine-rich repeat kinase 2 (LRRK)2*	*Microtubule-associated protein tau (MAPT)* *Bone marrow stromal cell antigen 1 (BST1)* *Major histocompatibility complex, class II, DR beta-1 (HLA-DRB1)* *Major histocompatibility complex, class II, DR beta-5 (HLA-DRB5)* *Transmembrane protein 175 (TMEM175)* *Solute carrier family 44, member 1 (SLC44A1)* *Alpha-2A-adrenergic receptor (ADRA2A)* *Cathepsin B (CTSB)* *Scavenger receptor class B, member 2 (SCARB2)* *Synaptic vesicle glycoprotein 2C (SV2C)* *Sterol regulatory element-binding transcription factor 1 (SREBF1)* *Cyclin G-associated kinase (GAK)* *Glycoprotein NMB (GPNMB)*	*Ubiquitin carboxyl-terminal esterase L1 (UCHL1)* *Rab7-like 1 (RAB29)*

Abbreviations: PD = Parkinson’s disease; GWAS = genome-wide association study.

**Table 2:** Demographics and clinical characteristics of PD patients and controls.

Number of PD patients	311
Male sex	170 (54.66%)
Positive family history	13 (4.18%)
Mean age	66.54 (±8.75)
Mean age of onset	60.51 (±9.75)
Young onset (<45 years)	16 (5.14%)
Mean disease duration	6.48 (±5.12)
LEDD	456.56 (±309.7)
MoCA	23.25 (±4.99)
H&Y stage	
1	19 (6.11%)
2	170 (54.66%)
3	72 (23.15%)
4	35 (11.25%)
5	15 (4.82%)
Number of healthy controls	699
Male sex	305 (44%)
Mean age	55.5 (±8.27)

Abbreviations: PD = Parkinson’s disease, LEDD = levodopa equivalent daily dose, MoCA = Montreal Cognitive Assessment, H&Y = Hoehn & Yahr.

**Table 3:** Clinical scores of motor, non-motor and cognitive function of PD patients (*n* = 311).

PD Clinical Scores		Mean	SD
MDS-UPDRS Part I		8.84	5.74
MDS-UPDRS Part II		13.38	7.87
MDS-UPDRS Part III		29.46	18.16
	Rigidity sub-score	3.27	3.31
	Bradykinesia sub-score	15.94	10.64
	Gait impairment sub-score	3.97	4.25
	Tremor sub-score	4.44	4.96
MDS-UPDRS Part IV			
	Dyskinesia	0.29	0.94
	Motor fluctuations	1.96	2.72
NMSQuest		10.49	4.87
	Neuropsychiatric	1.89	1.61
	Anxiety and depression	0.83	0.86
	Apathy	0.31	0.46
	Cognitive deficits and psychosis	0.76	0.80
	Olfactory deficits	0.39	0.49
	Pain	0.35	0.48
	Sleep disorder	2.22	1.32
	Autonomic dysfunction	4.39	2.11
MoCA ^†^		23.25	4.99

Abbreviations: PD = Parkinson’s disease, MDS-UPDRS = Movement Disorder Society-Sponsored Revision of the Unified Parkinson’s Disease Rating Scale, NMSQuest = Non-Motor Symptoms Questionnaire, MoCA = Montreal Cognitive Assessment. ^†^ Missing data from one subject.

**Table 4:** Rare variants associated with PD or related disorders on ClinVar.

Gene	Variant	Condition	Interpretation	Allele Frequency	
				PD (AC = 622)	Controls (AC = 1398)
*DNAJC6*	R282C	Juvenile onset PD 19A	Uncertain significance	0.0016	0
*FBXO7*	Y52C	Parkinsonian-pyramidal syndrome	Likely benign	0.0064	0.0029
*FBXO7*	R438C	Parkinsonian-pyramidal syndrome	Uncertain significance	0.0032	0.0028
*GBA1*	D448H ^†^	PD Gaucher disease	Pathogenic	0.0016	0
*GBA1*	R87W	PD Gaucher disease	Pathogenic	0.0016	0
*GBA1*	c.762-1G>C	PD Gaucher disease	Pathogenic	0.0032	0
*LRRK2*	A419V	Autosomal dominant PD 8	Likely benign	0.0048	0.0035
*LRRK2*	P755L	Autosomal dominant PD 8	Likely benign	0.0048	0.0071
*LRRK2*	L1388I	Autosomal dominant Parkinson disease 8	Likely benign	0.0016	0.0028
*LRRK2*	R1441C	Autosomal dominant Parkinson disease 8	Pathogenic	0.0016	0
*MAPT*	Q230R	Parkinson disease, late-onset	Likely benign	0.0161	0.0086
*MAPT*	R5H	Dementia, frontotemporal, with parkinsonism	Pathogenic	0.0032	0.0014
*MAPT*	S687L	Supranuclear palsy, progressive, 1	Pathogenic	0.0016	N/A
*PARK2*	C441R	Autosomal recessive juvenile PD 2	Likely pathogenic	0.0032	0.0007
*PARK2*	R366W	Autosomal recessive juvenile PD 2	Benign	0.0016	0.0014
*PINK1*	M341I	Autosomal recessive early-onset PD 6	Likely benign	0.0032	0.0064
*PINK1*	A280T	Autosomal recessive early-onset PD 6	Uncertain significance	0.0016	0.0014
*PINK1*	R492 *	Autosomal recessive early-onset PD 6	Pathogenic	0.0016	0.0007
*PLA2G6*	P806R	Autosomal recessive PD 14	Uncertain significance	0.011	0.0043
*PLA2G6*	c.1427+1G>A	Autosomal recessive PD 14	Likely pathogenic	0	0.0007
*SYNJ1*	Y400N	Early-onset PD 20	Benign	0.003	0
*SYNJ1*	A551V	Early-onset PD 20	Likely benign	0.0016	0.0043
*SYNJ1*	P1578L	Early-onset PD 20	Uncertain significance	0.0016	0

Abbreviations: PD = Parkinson’s disease; AC = allele count; N/A = not applicable, this variant was not analyzed in controls due to inadequate sequencing coverage in control samples. ^†^ Also known as D409H.

**Table 5:** Aggregate rare variant burden in patients and controls.

	PD (*n* = 311)	Controls (*n* = 699)
0 variant	89 (29%)	440 (63%)
1 variant	115 (37%)	195 (28%)
2 or more variants	107 (34%)	64 (9%)

Comparison was made between PD and control groups using chi-square test (*p* = 6.28 × 10^−75^).

**Table 6:** Correlation between rare variant burden of PD genes and clinical features.

Clinical Feature	Gene	Gene Product	*p*-Value ^†^	RV Carriers (Mean Score ^‡^ ± SD)	RV Non-Carriers (Mean Score ^‡^ ± SD)
Age of onset	*GBA*	Glucocerebrosidase	0.0235	58.5 y (median)	61 y (median)
Tremor	*HLA-DRB5*	HLA class II histocompatibility antigen, DR beta 5 chain	0.004	4.31 ± 4.96	4.98 ± 4.94
	*LRRK2*	Leucine-rich repeat kinase 2	0.037	6.50 ± 8.22	4.39 ± 4.88
Gait	*SREBF1*	Sterol regulatory element-binding protein 1	0.0326	6.14 ± 5.93	3.92 ± 4.20
Motor fluctuations	*SYNJ1*	Synaptojanin-1	0.0077	3.83 ± 4.75	1.92 ± 2.67
	*SCARB2*	Lysosomal integral membrane protein 2	0.0339	3.50 ± 3.55	1.92 ± 2.69
Dyskinesia	*PLA2G6* ^§^	Calcium-independent phospholipase A2 beta	0.00895	6	0.27 ± 0.88
Neuropsychiatric	*GBA*	Glucocerebrosidase	0.0343	3.00 ± 2.37	1.87 ± 1.59
Anxiety and depression	*SCARB2*	Lysosomal integral membrane protein 2	0.0416	1.38 ± 0.92	0.81 ± 0.86
Olfactory deficit	*SLC44A1*	Choline transporter-like protein 1	0.0096	1 ± 0.00	0.38 ± 0.49
Pain	*SYNJ1*	Synaptojanin-1	0.0139	0.67 ± 0.52	0.34 ± 0.47
	*LRRK2*	Leucine-rich repeat kinase 2	0.014	0.50 ± 0.55	0.34 ± 0.48
	*GPNMB*	Transmembrane glycoprotein NMB	0.0381	0.57 ± 0.53	0.34 ± 0.48
Sleep disorder	*PLA2G6* ^§^	Calcium-independent phospholipase A2 beta	0.0128	0	2.23 ± 1.32
	*SNCA* ^§^	Alpha-synuclein	0.0215	0	2.23 ± 1.32
Autonomic dysfunction	*HLA-DRB5*	HLA class II histocompatibility antigen, DR beta 5 chain	0.0338	4.44 ± 2.13	4.17 ± 2.04

Abbreviation: RV = rare variant. † Comparison was made using Sequencing Kernel Association Test (SKAT), with permutated *p*-values corrected for multiple comparisons. Statistical significance was defined as *p* < 0.05. ‡ Higher scores indicate increased severity or prevalence of symptom(s). ^§^ Only one variant carrier.

## Data Availability

The datasets used and/or analyzed during the current study are available from the corresponding author on reasonable request.

## Supplementary Materials

The following supporting information can be downloaded at: https://www.mdpi.com/article/10.3390/ijms27177653/s1.

## Abbreviations

The following abbreviations are used in this manuscript:

APC	Antigen-presenting cells
CADD	Combined Annotation Dependent Depletion
EOPD	Early-onset Parkinson’s disease
GWAS	Genome-wide association studies
LOFTEE	Loss-of-function Transcript Effect Estimator
MAF	Minor allele frequency
MDS-UPDRS	Movement Disorder Society-Sponsored Revision of the Unified Parkinson’s Disease Rating Scale
MHC	Major histocompatibility complex
MoCA	Montreal Cognitive Assessment
NMS	Non-motor symptoms
NMSQuest	Non-motor Symptoms Questionnaire
PD	Parkinson’s disease
QC	Quality control
RV	Rare variant
SD	Standard deviation
SKAT	Sequencing Kernel Association Test
SNP	Single nucleotide polymorphisms
VEP	Variant Effect Predictor
