# Supplementary material for: Rare Variants May Influence Disease Risk and Clinical Features in Sporadic Late-Onset Chinese Parkinson’s Disease Patients
Source: Int J Mol Sci. 2026 Aug 26;27(17):7653. doi: 10.3390/ijms27177653 (PMC13566319; doi:10.3390/ijms27177653)
Supplement: Supplementary file 1 [file ijms-27-07653-s001.zip › ijms-4510385-supplementary.pdf]

Table S1 Rare variants identified in PD patients and healthy controls.

Gene	Variant	Minor allele Frequency			p-value#
		PD (n=311)	Control (n=699)	gnomAD East Asia	
DJ1	p.Tyr67Cys	0.001607717	0	-	0.677196355
DJ1	p.Arg156Trp	0.001607717	0	5.80E-05	0.677196355
ATP13A2	c.3405+1G>A	0.001607717	0	-	0.677196355
ATP13A2	p.Ala1086Glu	0.001607717	0	0	0.677196355
ATP13A2^	p.Thr733Met	0.004823151	0.001430615	0.003827	0.35097086
ATP13A2	p.Pro730Leu	0.001607717	0	0	0.677196355
ATP13A2	p.Gly360Glu	0.001607717	0.004291845	0.002727	0.590219325
ATP13A2	p.Ala249Thr	0.001607717	0.00286123	0.001567	0.969319187
ATP13A2	p.Leu77Pro	0.001607717	0	-	0.677196355
PINK1	p.Ile224Thr	0.001607717	0	0.0001166	0.677196355
PINK1	p.Ala280Thr	0.001607717	0.001430615	0.0005798	1
PINK1	p.Met341Ile	0.003215434	0.006437768	0.002493	0.560162292
PINK1	p.Val350Leu	0.001607717	0	0.001218	0.677196355
PINK1	p.Asp375His	0.001607717	0	0.0001739	0.677196355
PINK1	p.Arg464Cys	0.001607717	0	-	0.677196355
PINK1	p.Arg492Ter	0.001607717	0.000715308	0.0005801	1
DNAJC6	p.Arg282Cys	0.001607717	0	0.0004639	0.677196355
DNAJC6	p.Phe667Cys	0.001607717	0	-	0.677196355
DNAJC6	p.His679Arg	0.001607717	0	0.0004387	0.677196355
GBA^	p.Ile511Ser	0.001607717	0	-	0.677196355
GBA	p.Thr510AsnfsTer54	0.001607717	0	0	0.677196355
GBA	p.Asp448His	0.001607717	0	-	0.677196355
GBA	p.Glu388Lys	0.001607717	0	-	0.677196355
GBA^	c.762-1G>C	0.003215434	0	-	0.175210037
GBA	p.Arg202Gln	0.001607717	0.001430615	0.002956	1
GBA^	c.588+1G>A	0.001607717	0	-	0.677763764

GBA	p.Arg87Trp	0.001607717	0	-	0.677196355
GBA	p.Asp63Asn	0.001607717	0	-	0.677196355
RAB7L1^	p.Gln25Arg	0	0.001430615	5.80E-05	0.859024424
GAK	p.Ser1300Leu	0.004823151	0.001430615	0.004416	0.35097086
GAK^	p.Pro1139Arg	0.004823151	0.005722461	0.003442	1
GAK	p.Phe981Tyr	0.001607717	0.000715308	-	1
GAK	p.Ala534Gly	0.001607717	0.003576538	0.001906	0.757890244
GAK	p.Val452Ile	0.001607717	0	0.0009857	0.677196355
GAK	p.Gly380Asp	0.001607717	0	-	0.677196355
GAK	p.Thr286Pro	0.001607717	0	0.0006411	0.677196355
TMEM175	p.Lys258Glu	0.001607717	0.000715308	0.00029	1
TMEM175	p.Pro288Leu	0.001607717	0	0.0006984	0.677196355
BST1^	p.Arg124Cys	0.006430868	0.006437768	0.01212	1
SCARB2	p.Asn437Ser	0.001607717	0	-	0.677196355
SCARB2	p.Thr398Met	0.003215434	0	0.0007538	0.175210037
SCARB2	p.Ile353Val	0.003215434	0.001430615	0.0007537	0.77090306
SCARB2^	p.Asp194Asn	0.004823151	0.000715308	0.002319	0.16868078
SCARB2	p.Thr172Ile	0.001607717	0.000715308	0.001276	1
SCARB2	p.Phe168Leu	0.001607717	0	0.001855	0.677196355
SCARB2	p.Ile144Leu	0.001607717	0.001430615	0.00429	1
SNCA	p.Met127Ile	0.001607717	0	0.001102	0.677196355
SV2C	p.Arg121Trp	0.003215434	0	0.001685	0.175210037
SV2C	p.Val510Ile	0.001607717	0	0.0006957	0.677196355
HLA-DRB5	p.Thr214MetfsTer17	0.22508039	N/A	-	N/A
HLA-DRB5	p.Thr192ArgfsTer12	0.0048231510	N/A	0.01209	N/A
HLA-DRB5	p.Val104ArgfsTer26	0.207395498	0	0.0004501	3.12155E-60*
HLA-DRB5	p.Asp99ThrfsTer30	0.17202572	N/A	0.0164	N/A
HLA-DRB5	p.Ile60ThrfsTer21	0.17845659	N/A	0.01286	N/A
HLA-DRB5	p.His57GlnfsTer5	0.17845659	N/A	0.01004	N/A

HLA-DRB5	p.Gly8TrpfsTer34	0.07073955	N/A	-	N/A
HLA-DRB1	p.Thr262Ala	0.003246753	N/A	0.0001595	N/A
HLA-DRB1	p.Ser208Asn	0.001607717	N/A	0.0006021	N/A
HLA-DRB1	p.Ser196MetfsTer14	0.21864952	N/A	0.003493	N/A
HLA-DRB1	p.Gln136SerfsTer13	0.21382637	N/A	0.0006017	N/A
HLA-DRB1	p.Thr135AspfsTer23	0.21864952	N/A	0.0005413	N/A
HLA-DRB1	p.Asp105Gly	0.004823151	N/A	-	N/A
HLA-DRB1	p.Ala102ArgfsTer25	0.14308682	N/A	0	N/A
HLA-DRB1	p.Arg101GlyfsTer29	0.001851852	N/A	0	N/A
HLA-DRB1	p.Gln99ArgfsTer31	0.249311688	N/A	0.04645	N/A
HLA-DRB1	c.101-1G>A	0.003215434	N/A	0	N/A
PRKN	p.Met458Leu	0.003215434	0.001430615	0.00302	0.77090306
PRKN	p.Cys441Arg	0.003215434	0.000715308	0.0006389	0.470445401
PRKN	p.Pro437Ala	0.001607717	0	0.0001162	0.677196355
PRKN	p.Arg366Trp	0.001607717	0.001430615	0.003189	1
PRKN	p.Leu176Ser	0.001607717	0	-	0.677196355
GPNMB	p.Arg189Ter	0.001607717	0.005007153	0.002261	0.458840661
GPNMB	p.Asp228Glu	0.001607717	0.000715308	0.000116	1
GPNMB	p.Val231Met	0.001607717	0	0.0002319	0.677196355
GPNMB	p.Arg248Gln	0.003215434	0.000715308	-	0.470445401
GPNMB	p.Asp252Asn	0.001607717	0.003576538	0.00429	0.775206791
GPNMB	p.Ser484Ala	0.001607717	0	5.80E-05	0.677196355
CTSB^	p.Asp317Asn	0.006430868	0.020028612	0.02459	0.037222994
CTSB	p.His224Asp	0.001607717	0	0.0001293	0.677196355
CTSB	p.Arg195Trp	0.001607717	0	-	0.677196355
CTSB	p.Val177Ile	0.001607717	0.004291845	0.006613	0.590219325
CTSB	p.Gly147Glu	0.001607717	0	-	0.677196355
CTSB	p.Gly64Ala	0.001607717	0.000715308	0.000116	1
SLC44A1	p.Arg105Gln	0.001607717	0.00286123	0.004818	0.969319187

SLC44A1	p.Asp284Val	0.001607717	0.000715308	-	1
SLC44A1	p.Ser644Ala	0.003215434	0	0.0003545	0.175210037
ADRA2A	p.Arg3Cys	0.001607717	0	0.003245	0.76248654
ADRA2A	p.Glu155Lys	0.003215434	0.005722461	0.001798	0.690079401
ADRA2A	p.Arg202Leu	0.001607717	0	0.0007268	0.677196355
ADRA2A	p.Pro251Arg	0.001607717	0.004291845	0.005121	0.590219325
ADRA2A	p.Arg378Cys	0.001607717	0	-	0.677196355
LRRK2	p.Ala419Val	0.004823151	0.003576538	0.004929	0.977527706
LRRK2^	p.Pro755Leu	0.004823151	0.007153076	0.009297	0.774480531
LRRK2	p.Leu1388Ile	0.001607717	0.00286123	0.00238	0.969319187
LRRK2	p.Arg1441Cys	0.001607717	0	0	0.677196355
LRRK2	p.Val2526Ala	0.001607717	0	-	0.677196355
VPS13C	p.Gly3695Asp	0.003215434	0.003576538	0.002956	1
VPS13C^	p.Gly3616Asp	0.012861736	0.012875536	0.0205	1
VPS13C	p.Phe3427Leu	0.003215434	0	-	0.175210037
VPS13C	p.Met3413Leu	0.003215434	0	-	0.175210037
VPS13C	p.Leu3311Ter	0.001607717	0	-	0.677196355
VPS13C	p.Asn3100Ser	0.001607717	0	-	0.677196355
VPS13C	p.Arg3058Cys	0.001607717	0.004291845	0.001044	0.590219325
VPS13C	p.Ile2952Val	0.001607717	0	-	0.677196355
VPS13C	p.Arg2785His	0.003215434	0.001430615	0.0002899	0.77090306
VPS13C	p.Ala2520Gly	0.001607717	0	0.0006692	0.677196355
VPS13C	p.Val2426Ile	0.001607717	0	0	0.677196355
VPS13C^	p.Ile2081Phe	0.004823151	0.002145923	0.003131	0.562763419
VPS13C^	p.Ala2043Gly	0.004823151	0.005007153	0.004757	1
VPS13C	p.Ile1664Val	0.001607717	0.001430615	0.0018	1
VPS13C	p.Ile1619Thr	0.001607717	0.000715308	0.0007969	1
VPS13C	p.Thr1522Ser	0.003215434	0.004291845	0.004066	1
VPS13C	p.Glu1055Ala	0.003215434	0.004291845	0.002796	1

VPS13C	c.2911+2T>C	0.001607717	0.000715308	-	1
VPS13C	p.Ser874Ala	0.001607717	0.000715308	0.0002917	1
VPS13C^	p.Leu866Ser	0.004823151	0.003576538	0.003189	0.977527706
VPS13C^	p.Arg800Ser	0.012861736	0.014306152	0.0104	0.800355747
VPS13C	p.Tyr746His	0.001607717	0	0.0001162	0.677196355
VPS13C^	p.Asp622Glu	0.006430868	0.007153076	0.007382	1
VPS13C	p.Lys574Glu	0.001607717	0	5.84E-05	0.677196355
VPS13C	p.Ser515Arg	0.001607717	0	-	0.677196355
VPS13C	p.Gln447Leu	0.003215434	0	0.0001194	0.175210037
VPS13C	p.Gln446His	0.001607717	0	0.0003577	0.677196355
VPS13C^	p.Asp172Gly	0.004823151	0.002145923	0.002094	0.562763419
VPS13C	p.Lys158Gln	0.001607717	0	-	0.677196355
VPS13C^	p.Arg153Cys	0.009646302	0.004291845	0.004761	0.256181713
VPS13C	p.His131Arg	0.001607717	0.000715308	0.002378	1
VPS13C	p.Leu88Val	0.001607717	0.000715308	0.0002383	1
VPS13C	p.Tyr87Asn	0.001607717	0	-	0.677763764
POLG	p.Asp1219Gly	0.001607717	0	0	0.677196355
POLG	p.Ala962Thr	0.001607717	0.000715308	0.0004638	1
POLG	p.Tyr614His	0.001607717	0.000715308	0.0004638	1
POLG	p.Leu311Gln	0.001607717	0	-	0.677196355
POLG	p.Leu244Phe	0.001607717	0	-	0.677196355
POLG	p.Ser29Cys	0.001607717	0.001430615	0.0001786	1
POLG	p.Pro22Ser	0.001607717	0.000715308	9.76E-05	1
SREBF1^	p.Ala924Val	0.008038585	0.005007153	0.003795	0.612589902
SREBF1	p.Asn823Ser	0.001607717	0.000715308	0.001397	1
SREBF1	p.Thr522Met	0.001607717	0.000715308	0.0004227	1
SREBF1	p.Gly317Ser	0.001607717	0.000715308	0.0006957	1
SREBF1	p.Pro310Leu	0.001607717	0	-	0.677196355
SREBF1	p.Pro223Leu	0.003215434	0.001430615	0.0004666	0.77090306

SREBF1	p.Lys138Asn	0.001607717	0	0.0002319	0.677196355
MAPT	p.Arg5His	0.003215434	0.001430615	0.0006379	0.77090306
MAPT	p.Gly60Ser	0.001607717	0	-	0.677196355
MAPT	p.Pro140Ser	0.003215434	0.007153076	0.007022	0.452180755
MAPT^	p.Asp177Val	0.004823151	0.001430615	0.001255	0.35097086
MAPT^	p.Gln230Arg	0.01607717	0.008583691	0.01542	0.20309096
MAPT	p.Arg364Trp	0.001607717	0	0	0.677196355
MAPT	p.Gly539Ser	0.001607717	0	-	0.677196355
MAPT	p.Ser687Leu	0.001607717	N/A	-	N/A
FBXO7^	p.Tyr52Cys	0.006430868	0.00295421	0.004754	0.140918286
FBXO7	p.Ser150Leu	0.001607717	0	5.80E-05	0.677196355
FBXO7	p.Arg432Met	0.001607717	0.000715308	-	1
FBXO7	p.Arg438Cys	0.003215434	0.00286123	0.0005218	1
FBXO7	p.Pro496Thr	0.001607717	0	0.0004058	0.677196355
PLA2G6^	p.Pro806Arg	0.011254019	0.004291845	0.01008	0.1310516
PLA2G6^	p.Thr575Met	0	0.000715308	0.0003479	1
PLA2G6^	p.Leu491Val	0	0.000715308	-	1
PLA2G6^	c.1427+1G>A	0	0.000715308	0.000116	1
PLA2G6^	p.Thr387Arg	0	0.000715308	-	1
PLA2G6^	p.Asp331Tyr	0	0.000715308	0.0006361	1
PLA2G6^	p.Gly266Ala	0	0.000715308	-	1
PLA2G6^	p.Tyr254Cys	0	0.000715308	-	1
PLA2G6^	p.Glu133Lys	0	0.000715308	5.93E-05	1
PLA2G6	p.Arg53His	0.001607717	0.002145923	0.000174	1
PLA2G6^	p.Asn16Ser	0	0.000715308	0	1
PLA2G6^	p.Phe3Leu	0	0.000715308	-	1
SYNJ1	p.Pro1578Leu	0.001607717	0	-	0.677196355
SYNJ1	p.Ala551Val	0.001607717	0.004291845	0.004685	0.590219325
SYNJ1	p.Tyr400Asn	0.003215434	0	0.002537	0.175210037

SYNJ1	p.Ala383Gly	0.001607717	0.000715308	-	1
SYNJ1	p.Ala240Thr	0.001607717	0	0.0003479	0.677196355
gnomAD = Genome Aggregation Database. # = Chi-square test. * p-value< 0.0003. ^ = These variants were not included in the phenotype correlation analysis within PD patients due to inadequate sequencing coverage in PD group or they were not present in the PD group. - = not reported in gnomAD East Asian population. N/A = Not applicable, these variants have not been analyzed in controls due to inadequate sequencing coverage in control group.					

Table S2 Rare variants listed by PD patients.

PD ID	Number of variants	Rare variants
PD001	1	HLA_DRB5_p_Val104ArgfsTer26
PD002	0	
PD003	1	HLA_DRB5_p_Val104ArgfsTer26
PD004	0	
PD005	3	HLA_DRB5_p_Val104ArgfsTer26, LRRK2_p_Val2526Ala, VPS13C_p_Arg800Ser
PD006	2	HLA_DRB5_p_Val104ArgfsTer26, VPS13C_p_Tyr746His
PD007	1	SLC44A1_p_Asp284Val
PD008	0	
PD009	2	POLG_p_Leu311Gln, MAPT_p_Pro140Ser
PD010	1	ADRA2A_p_Arg3Cys
PD011	1	VPS13C_p_Arg800Ser
PD012	1	PLA2G6_p_Pro806Arg
PD014	1	HLA_DRB5_p_Val104ArgfsTer26
PD015	2	POLG_p_Ser29Cys, PLA2G6_p_Pro806Arg
PD016	2	HLA_DRB5_p_Val104ArgfsTer26, HLA_DRB5_p_Val104ArgfsTer26
PD017	0	
PD018	1	HLA_DRB5_p_Val104ArgfsTer26
PD019	1	PINK1_p_Arg464Cys
PD020	3	HLA_DRB5_p_Val104ArgfsTer26, VPS13C_p_Arg2785His, VPS13C_p_Gln447Leu
PD021	1	HLA_DRB5_p_Val104ArgfsTer26
PD022	3	HLA_DRB5_p_Val104ArgfsTer26, VPS13C_p_Gly3616Asp, POLG_p_Pro22Ser
PD023	2	HLA_DRB5_p_Val104ArgfsTer26, PARK2_p_Pro437Ala
PD024	1	BST1_p_Arg124Cys
PD025	2	HLA_DRB5_p_Val104ArgfsTer26, FBXO7_p_Tyr52Cys
PD026	1	VPS13C_p_Arg3058Cys
PD027	0	
PD028	2	GAK_p_Ser1300Leu, HLA_DRB5_p_Val104ArgfsTer26
PD030	0	
PD031	0	

PD032	1	HLA_DRB5_p_Val104ArgfsTer26
PD033	2	GAK_p_Ser1300Leu, VPS13C_p_Asp172Gly
PD034	1	HLA_DRB5_p_Val104ArgfsTer26
PD035	1	VPS13C_p_Arg800Ser
PD036	2	ATP13A2_p_Pro730Leu, HLA_DRB5_p_Val104ArgfsTer26
PD037	1	HLA_DRB5_p_Val104ArgfsTer26
PD038	1	VPS13C_p_Ile2952Val
PD039	2	VPS13C_p_Met3413Leu, MAPT_p_Arg5His
PD040	0	
PD041	0	
PD042	2	GBA_p_Asp448His, HLA_DRB5_p_Val104ArgfsTer26
PD043	1	FBXO7_p_Arg438Cys
PD045	2	HLA_DRB5_p_Val104ArgfsTer26, VPS13C_p_Lys574Glu
PD046	3	HLA_DRB5_p_Val104ArgfsTer26, VPS13C_p_Arg153Cys, PLA2G6_p_Pro806Arg
PD047	1	SREBF1_p_Gly317Ser
PD048	0	
PD049	1	VPS13C_p_Leu3311Ter
PD050	0	
PD051	0	
PD052	2	HLA_DRB5_p_Val104ArgfsTer26, MAPT_p_Gln230Arg
PD053	1	MAPT_p_Arg5His
PD054	3	HLA_DRB5_p_Val104ArgfsTer26, HLA_DRB5_p_Val104ArgfsTer26, GPNMB_p_Arg248Gln
PD055	0	
PD056	0	
PD057	2	HLA_DRB5_p_Val104ArgfsTer26, GPNMB_p_Arg189Ter
PD058	0	
PD059	2	PARK2_p_Met458Leu, MAPT_p_Pro140Ser
PD060	1	DJ1_p_Tyr67Cys
PD061	1	ATP13A2_p_Ala249Thr
PD062	3	GBA_p_Arg202Gln, TMEM175_p_Lys258Glu, HLA_DRB5_p_Val104ArgfsTer26
PD063	1	SCARB2_p_Asn437Ser

PD064	1	DNAJC6_p_His679Arg
PD065	0	
PD066	0	
PD067	1	BST1_p_Arg124Cys
PD068	0	
PD069	1	VPS13C_p_Gly3695Asp
PD070	1	BST1_p_Arg124Cys
PD071	2	PINK1_p_Met341Ile, CTSB_p_Asp317Asn
PD072	3	DJ1_p_Arg156Trp, GAK_p_Pro1139Arg, HLA_DRB5_p_Val104ArgfsTer26
PD073	3	PINK1_p_Ile224Thr, HLA_DRB5_p_Val104ArgfsTer26, MAPT_p_Gln230Arg
PD074	3	HLA_DRB5_p_Val104ArgfsTer26, CTSB_p_His224Asp, VPS13C_p_Arg2785His
PD075	0	
PD076	1	HLA_DRB5_p_Val104ArgfsTer26
PD077	0	
PD078	4	HLA_DRB5_p_Val104ArgfsTer26, ADRA2A_p_Arg202Leu, VPS13C_p_Arg153Cys, SREBF1_p_Thr522Met
PD079	1	HLA_DRB5_p_Val104ArgfsTer26
PD080	2	HLA_DRB5_p_Val104ArgfsTer26, HLA_DRB5_p_Val104ArgfsTer26
PD081	0	
PD082	0	
PD083	2	SCARB2_p_Asp194Asn, VPS13C_p_Gly3616Asp
PD084	0	
PD085	2	GAK_p_Pro1139Arg, ADRA2A_p_Glu155Lys
PD086	0	
PD087	0	
PD088	1	VPS13C_p_Asn3100Ser
PD089	0	
PD090	0	
PD091	2	SNCA_p_Met127Ile, VPS13C_p_Asp622Glu
PD092	2	SV2C_p_Val510Ile, MAPT_p_Gly539Ser
PD093	2	SREBF1_p_Ala924Val, MAPT_p_Gln230Arg
PD094	1	SYNJ1_p_Ala551Val

PD095	0	
PD096	3	PINK1_p_Ala280Thr, HLA_DRB5_p_Val104ArgfsTer26, SREBF1_p_Asn823Ser
PD097	1	HLA_DRB5_p_Val104ArgfsTer26
PD098	1	HLA_DRB5_p_Val104ArgfsTer26
PD099	1	HLA_DRB5_p_Val104ArgfsTer26
PD100	1	ATP13A2_p_Thr733Met
PD101	1	LRRK2_p_Arg1441Cys
PD102	1	HLA_DRB5_p_Val104ArgfsTer26
PD103	1	MAPT_p_Asp177Val
PD104	1	HLA_DRB5_p_Val104ArgfsTer26
PD105	1	SCARB2_p_Ile353Val
PD106	1	HLA_DRB5_p_Val104ArgfsTer26
PD107	3	VPS13C_p_Gly3616Asp, VPS13C_p_Ile1664Val, MAPT_p_Asp177Val
PD108	1	VPS13C_p_Arg153Cys
PD109	0	
PD110	1	FBXO7_p_Pro496Thr
PD111	0	
PD112	2	HLA_DRB5_p_Val104ArgfsTer26, MAPT_p_Gln230Arg
PD113	0	
PD114	1	SLC44A1_p_Ser644Ala
PD115	0	
PD116	2	ATP13A2_p_Gly360Glu, HLA_DRB5_p_Val104ArgfsTer26
PD117	0	
PD118	1	PINK1_p_Arg492Ter
PD119	2	HLA_DRB5_p_Val104ArgfsTer26, VPS13C_p_Gly3695Asp
PD120	2	HLA_DRB5_p_Val104ArgfsTer26, FBXO7_p_Arg432Met
PD121	2	HLA_DRB5_p_Val104ArgfsTer26, CTSB_p_Asp317Asn
PD122	1	VPS13C_p_Ile1619Thr
PD123	1	HLA_DRB5_p_Val104ArgfsTer26
PD124	1	FBXO7_p_Tyr52Cys
PD125	2	DNAJC6_p_Arg282Cys, SREBF1_p_Pro223Leu

PD126	0	
PD127	2	HLA_DRB5_p_Val104ArgfsTer26, SYNJ1_p_Tyr400Asn
PD128	1	SLC44A1_p_Ser644Ala
PD129	1	HLA_DRB5_p_Val104ArgfsTer26
PD13	1	GBA_c_588+1G>A
PD130	1	MAPT_p_Asp177Val
PD131	1	HLA_DRB5_p_Val104ArgfsTer26
PD132	0	
PD133	0	
PD134	0	
PD135	1	SREBF1_p_Pro310Leu
PD136	1	SCARB2_p_Thr172Ile
PD137	0	
PD138	1	VPS13C_p_Val2426Ile
PD139	4	HLA_DRB5_p_Val104ArgfsTer26, HLA_DRB5_p_Val104ArgfsTer26, VPS13C_p_Met3413Leu, SYNJ1_p_Ala383Gly
PD140	0	
PD141	3	VPS13C_p_Glu1055Ala, VPS13C_p_Asp622Glu, VPS13C_p_Lys158Gln
PD142	1	HLA_DRB5_p_Val104ArgfsTer26
PD143	1	HLA_DRB5_p_Val104ArgfsTer26
PD144	0	
PD145	1	ATP13A2_p_Leu77Pro
PD146	1	GBA_p_Arg87Trp
PD147	0	
PD148	0	
PD149	0	
PD150	1	HLA_DRB5_p_Val104ArgfsTer26
PD151	0	
PD152	2	GPNMB_p_Arg248Gln, LRRK2_p_Ala419Val
PD153	2	SCARB2_p_Ile353Val, VPS13C_p_Gly3616Asp
PD154	0	
PD155	0	

PD156	2	GAK_p_Ser1300Leu, SV2C_p_Arg121Trp
PD157	3	HLA_DRB5_p_Val104ArgfsTer26, ADRA2A_p_Arg378Cys, MAPT_p_Gln230Arg
PD158	0	
PD159	3	HLA_DRB5_p_Val104ArgfsTer26, LRRK2_p_Leu1388Ile, VPS13C_p_Ala2043Gly
PD160	2	HLA_DRB5_p_Val104ArgfsTer26, VPS13C_p_Leu866Ser
PD161	3	HLA_DRB5_p_Val104ArgfsTer26, VPS13C_p_Gly3616Asp, VPS13C_p_Arg800Ser
PD162	2	HLA_DRB5_p_Val104ArgfsTer26, VPS13C_p_Gln447Leu
PD163	2	HLA_DRB5_p_Val104ArgfsTer26, HLA_DRB5_p_Val104ArgfsTer26
PD164	3	HLA_DRB5_p_Val104ArgfsTer26, VPS13C_p_Ile2081Phe, FBXO7_p_Tyr52Cys
PD165	1	VPS13C_p_Asp622Glu
PD166	1	CTSB_p_Asp317Asn
PD167	2	SCARB2_p_Ile144Leu, MAPT_p_Gln230Arg
PD168	0	
PD169	2	DNAJC6_p_Phe667Cys, POLG_p_Asp1219Gly
PD170	0	
PD171	2	HLA_DRB5_p_Val104ArgfsTer26, HLA_DRB5_p_Val104ArgfsTer26
PD172	2	GPNMB_p_Asp252Asn, POLG_p_Leu244Phe
PD173	2	ATP13A2_p_Thr733Met, GBA_c_762_1G>C
PD174	1	HLA_DRB5_p_Val104ArgfsTer26
PD175	1	HLA_DRB5_p_Val104ArgfsTer26
PD176	0	
PD177	1	HLA_DRB5_p_Val104ArgfsTer26
PD178	0	
PD179	1	HLA_DRB5_p_Val104ArgfsTer26
PD180	3	ATP13A2_p_Ala1086Glu, HLA_DRB5_p_Val104ArgfsTer26, HLA_DRB5_p_Val104ArgfsTer26
PD181	1	VPS13C_p_Gly3616Asp
PD182	2	HLA_DRB5_p_Val104ArgfsTer26, VPS13C_p_His131Arg
PD183	1	HLA_DRB5_p_Val104ArgfsTer26
PD184	1	MAPT_p_Gln230Arg
PD185	2	VPS13C_p_Ala2043Gly, PLA2G6_p_Pro806Arg
PD186	0	

PD187	1	VPS13C_p_Arg153Cys
PD188	1	HLA_DRB5_p_Val104ArgfsTer26
PD189	2	GBA_p_Glu388Lys, HLA_DRB5_p_Val104ArgfsTer26
PD190	1	HLA_DRB5_p_Val104ArgfsTer26
PD191	0	
PD192	1	HLA_DRB5_p_Val104ArgfsTer26
PD193	2	CTSB_p_Gly147Glu, PLA2G6_p_Arg53His
PD194	0	
PD195	1	SCARB2_p_Asp194Asn
PD196	0	
PD197	1	LRRK2_p_Pro755Leu
PD198	0	
PD199	0	
PD200	0	
PD201	3	VPS13C_p_Glu1055Ala, VPS13C_p_Asp622Glu, PLA2G6_p_Pro806Arg
PD202	2	SV2C_p_Arg121Trp, HLA_DRB5_p_Val104ArgfsTer26
PD203	0	
PD204	1	HLA_DRB5_p_Val104ArgfsTer26
PD205	1	PINK1_p_Met341Ile
PD206	0	
PD207	1	HLA_DRB5_p_Val104ArgfsTer26
PD208	0	
PD210	0	
PD211	1	HLA_DRB5_p_Val104ArgfsTer26
PD212	3	HLA_DRB5_p_Val104ArgfsTer26, VPS13C_p_Leu866Ser, VPS13C_p_Arg800Ser
PD213	2	HLA_DRB5_p_Val104ArgfsTer26, SYNJ1_p_Pro1578Leu
PD214	0	
PD215	1	VPS13C_c_2911+2T>C
PD216	2	HLA_DRB5_p_Val104ArgfsTer26, VPS13C_p_Ala2043Gly
PD217	2	HLA_DRB5_p_Val104ArgfsTer26, VPS13C_p_Thr1522Ser
PD218	1	FBXO7_p_Arg438Cys

PD219	1	HLA_DRB5_p_Val104ArgfsTer26
PD220	1	HLA_DRB5_p_Val104ArgfsTer26
PD221	2	CTSB_p_Gly64Ala, SREBF1_p_Ala924Val
PD222	1	LRRK2_p_Ala419Val
PD223	2	GBA_p_Asp63Asn, TMEM175_p_Pro288Leu
PD224	0	
PD225	2	HLA_DRB5_p_Val104ArgfsTer26, MAPT_p_Gln230Arg
PD226	0	
PD227	2	SCARB2_p_Thr398Met, LRRK2_p_Pro755Leu
PD228	2	HLA_DRB5_p_Val104ArgfsTer26, VPS13C_p_Leu866Ser
PD229	0	
PD230	2	HLA_DRB5_p_Val104ArgfsTer26, VPS13C_p_Phe3427Leu
PD231	0	
PD232	0	
PD233	1	HLA_DRB5_p_Val104ArgfsTer26
PD234	1	SYNJ1_p_Ala240Thr
PD235	0	
PD236	1	VPS13C_p_Asp172Gly
PD237	0	
PD238	1	HLA_DRB5_p_Val104ArgfsTer26
PD239	1	HLA_DRB5_p_Val104ArgfsTer26
PD240	1	HLA_DRB5_p_Val104ArgfsTer26
PD241	2	SREBF1_p_Ala924Val, MAPT_p_Arg364Trp
PD242	2	HLA_DRB5_p_Val104ArgfsTer26, MAPT_p_Gln230Arg
PD243	3	GBA_p_Thr510AsnfsTer54, HLA_DRB5_p_Val104ArgfsTer26, PLA2G6_p_Pro806Arg
PD244	2	SCARB2_p_Thr398Met, HLA_DRB5_p_Val104ArgfsTer26
PD245	0	
PD246	2	SCARB2_p_Asp194Asn, LRRK2_p_Pro755Leu
PD247	1	GPNMB_p_Asp228Glu
PD248	1	ATP13A2_p_Thr733Met
PD249	1	PLA2G6_p_Pro806Arg

PD250	2	GAK_p_Val452Ile, POLG_p_Tyr614His
PD252	1	HLA_DRB5_p_Val104ArgfsTer26
PD253	0	
PD254	4	HLA_DRB5_p_Val104ArgfsTer26, SLC44A1_p_Arg105Gln, LRRK2_p_Ala419Val, VPS13C_p_Ile2081Phe
PD255	1	VPS13C_p_Phe3427Leu
PD256	0	
PD257	2	HLA_DRB5_p_Val104ArgfsTer26, VPS13C_p_Gln446His
PD258	2	GBA_c_762_1G>C, POLG_p_Ala962Thr
PD259	3	VPS13C_p_Thr1522Ser, VPS13C_p_Ser515Arg, VPS13C_p_Tyr87Asn
PD260	2	HLA_DRB5_p_Val104ArgfsTer26, SREBF1_p_Lys138Asn
PD261	1	HLA_DRB5_p_Val104ArgfsTer26
PD262	2	HLA_DRB5_p_Val104ArgfsTer26, MAPT_p_Gln230Arg
PD263	3	HLA_DRB5_p_Val104ArgfsTer26, HLA_DRB5_p_Val104ArgfsTer26, VPS13C_p_Arg153Cys
PD264	1	HLA_DRB5_p_Val104ArgfsTer26
PD265	1	HLA_DRB5_p_Val104ArgfsTer26
PD266	1	HLA_DRB5_p_Val104ArgfsTer26
PD267	0	
PD268	0	
PD269	0	
PD270	2	HLA_DRB5_p_Val104ArgfsTer26, SREBF1_p_Ala924Val
PD271	8	PINK1_p_Asp375His, GAK_p_Thr286Pro, HLA_DRB5_p_Val104ArgfsTer26, PARK2_p_Met458Leu, PARK2_p_Arg366Trp, GPNMB_p_Val231Met, VPS13C_p_Leu88Val, SYNJ1_p_Tyr400Asn
PD272	0	
PD273	2	VPS13C_p_Gly3616Asp, VPS13C_p_Ser874Ala
PD274	1	ADRA2A_p_Glu155Lys
PD275	0	
PD276	2	HLA_DRB5_p_Val104ArgfsTer26, SREBF1_p_Pro223Leu
PD277	1	HLA_DRB5_p_Val104ArgfsTer26
PD278	0	
PD279	2	PARK2_p_Leu176Ser, VPS13C_p_Arg800Ser
PD280	4	GBA_p_Ile511Ser, HLA_DRB5_p_Val104ArgfsTer26, MAPT_p_Gly60Ser, FBXO7_p_Ser150Leu

PD281	2	HLA_DRB5_p_Val104ArgfsTer26, GPNMB_p_Ser484Ala
PD282	1	GAK_p_Pro1139Arg
PD283	1	HLA_DRB5_p_Val104ArgfsTer26
PD284	1	HLA_DRB5_p_Val104ArgfsTer26
PD285	2	GAK_p_Gly380Asp, HLA_DRB5_p_Val104ArgfsTer26
PD286	3	PINK1_p_Val350Leu, HLA_DRB5_p_Val104ArgfsTer26, VPS13C_p_Gly3616Asp
PD287	0	
PD288	1	HLA_DRB5_p_Val104ArgfsTer26
PD289	3	SCARB2_p_Phe168Leu, PARK2_p_Cys441Arg, SREBF1_p_Ala924Val
PD29	0	
PD290	0	
PD291	1	CTSB_p_Val177Ile
PD292	1	CTSB_p_Asp317Asn
PD293	2	HLA_DRB5_p_Val104ArgfsTer26, PARK2_p_Cys441Arg
PD294	0	
PD295	2	HLA_DRB5_p_Val104ArgfsTer26, VPS13C_p_Arg153Cys
PD296	2	BST1_p_Arg124Cys, VPS13C_p_Ile2081Phe
PD297	2	GAK_p_Ala534Gly, HLA_DRB5_p_Val104ArgfsTer26
PD298	1	GAK_p_Phe981Tyr
PD299	2	HLA_DRB5_p_Val104ArgfsTer26, FBXO7_p_Tyr52Cys
PD300	0	
PD301	0	
PD302	2	ATP13A2_c_3405+1G>A, HLA_DRB5_p_Val104ArgfsTer26
PD303	1	VPS13C_p_Asp172Gly
PD304	0	
PD305	1	HLA_DRB5_p_Val104ArgfsTer26
PD306	1	HLA_DRB5_p_Val104ArgfsTer26
PD307	0	
PD308	0	
PD310	2	HLA_DRB5_p_Val104ArgfsTer26, VPS13C_p_Ala2520Gly
PD311	2	HLA_DRB5_p_Val104ArgfsTer26, VPS13C_p_Arg800Ser

PD312	1	ADRA2A_p_Pro251Arg
PD313	0	
PD314	2	HLA_DRB5_p_Val104ArgfsTer26, VPS13C_p_Arg800Ser
PD315	1	CTSB_p_Arg195Trp
